# Alteration of cholesterol distribution at the plasma membrane of cancer cells: From evidence to pathophysiological implication and promising therapy strategy

**DOI:** 10.3389/fphys.2022.999883

**Published:** 2022-11-09

**Authors:** Mauriane Maja, Donatienne Tyteca

**Affiliations:** CELL Unit, de Duve Institute, Université catholique de Louvain, Brussels, Belgium

**Keywords:** lipid rafts, submicrometric domains, caveolae, cancer, cell proliferation, apoptosis, cell migration, anticancer therapy

## Abstract

Cholesterol-enriched domains are nowadays proposed to contribute to cancer cell proliferation, survival, death and invasion, with important implications in tumor progression. They could therefore represent promising targets for new anticancer treatment. However, although diverse strategies have been developed over the years from directly targeting cholesterol membrane content/distribution to adjusting sterol intake, all approaches present more or less substantial limitations. Those data emphasize the need to optimize current strategies, to develop new specific cholesterol-targeting anticancer drugs and/or to combine them with additional strategies targeting other lipids than cholesterol. Those objectives can only be achieved if we first decipher (i) the mechanisms that govern the formation and deformation of the different types of cholesterol-enriched domains and their interplay in healthy cells; (ii) the mechanisms behind domain deregulation in cancer; (iii) the potential generalization of observations in different types of cancer; and (iv) the specificity of some alterations in cancer vs*.* non-cancer cells as promising strategy for anticancer therapy. In this review, we will discuss the current knowledge on the homeostasis, roles and membrane distribution of cholesterol in non-tumorigenic cells. We will then integrate documented alterations of cholesterol distribution in domains at the surface of cancer cells and the mechanisms behind their contribution in cancer processes. We shall finally provide an overview on the potential strategies developed to target those cholesterol-enriched domains in cancer therapy.

## 1 Introduction

As a ubiquitous sterol found in vertebrate organisms, cholesterol (chol) exerts pleiotropic biological actions in cell physiology. From maintaining the structural integrity and regulating the biophysical properties of the plasma membrane (PM) to serving as a precursor for steroid hormones, vitamin D and oxysterols, chol is involved at many subcellular levels. Therefore, its homeostasis must be tightly regulated as any disbalance could lead to cancer development. The metabolism of chol, trafficking and its related intracellular functions have been the subject of many investigation over the years (Ikonen 2008; Afonso et al., 2018; Luo et al., 2020). However, despite several years of research, whether this lipid plays a role in oncogenesis is still an open question. This could result from differential sometimes contradictory association between chol levels and different types of cancers ((Asano et al., 2008; Llaverias et al., 2011; Pelton et al., 2012; Murai 2015; Heir et al., 2016; Radisauskas et al., 2016); reviewed in (Vona et al., 2021)). In addition, although chol-enriched domains are nowadays proposed to contribute to cancer cell proliferation, survival, death and invasion with important implications in tumor progression, how those domains are modified in malignant cells remains poorly understood. Another concern is whether and how the PM transversal distribution of chol is also impaired in malignant cells and whether it is coupled to chol-enriched domain alteration. These are key questions that need to be answered before we move forward implementing a chol-enriched domain-mediated approach. In the present review we discuss the current knowledge on the homeostasis, roles and membrane distribution of chol in non-tumorigenic cells. We then integrate documented alterations of chol distribution in domains at the surface of cancer cells and the mechanisms behind their contribution in cancer processes. We finally provide an overview on the potential strategies developed to target chol-enriched domains in cancer therapy.

## 2 Physiological cholesterol homeostasis, roles and membrane distribution

In this section we summarize the key findings regarding chol homeostasis (Section 2.1), physiological roles (Section 2.2) and membrane distribution (Section 2.3) in non-tumorigenic cells.

### 2.1 Cholesterol homeostasis

**FIGURE 1:**
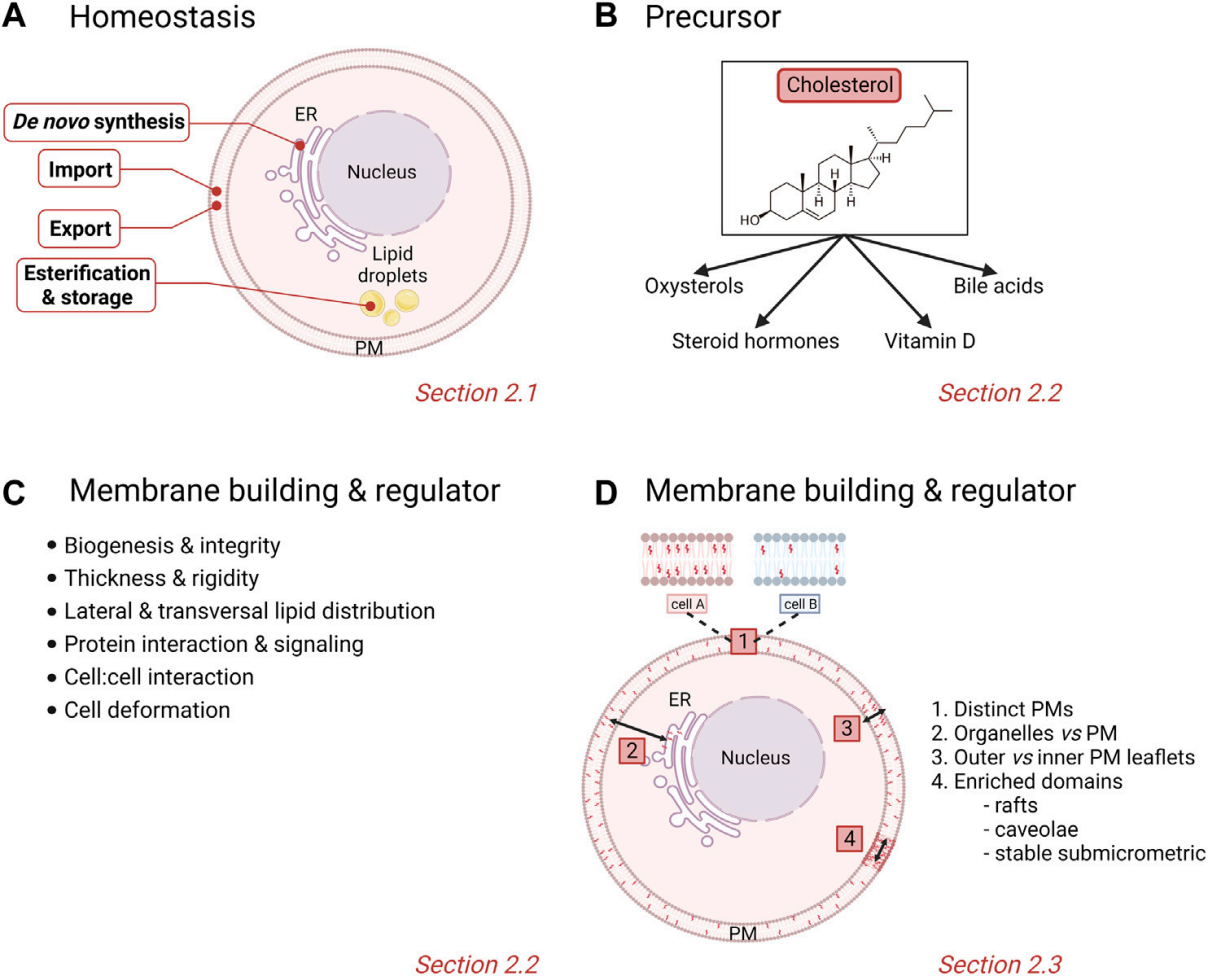
Physiological homeostasis, roles and cellular/membrane distribution of cholesterol. **(A)** Stages of chol homeostasis involving de novo synthesis, import, export, esterification and storage. **(B,C)** Pleiotropic actions of chol. **(D)** Differential levels of heterogeneity of membrane chol distribution in cells. PM, plasma membrane. See the Section 2 of the text for further details.

In normal cells, the metabolism of chol is tightly regulated and crucial for cellular integrity and biological functions. Any dysregulation in one or many stages of the chol homeostasis (import, synthesis, export and esterification) has been associated with pathological conditions such as cardiovascular disease, atherosclerosis and cancer. Nucleated cells use their endoplasmic reticulum (ER) chol levels as sensors to control the intracellular chol homeostasis. Simply, decreased ER chol levels activate sterol regulatory element binding proteins (SREBPs) that increase the transcription of genes involved in chol synthesis and import into cells. Conversely, increased intracellular chol levels activate another nuclear receptor system, the liver X receptors (LXRs) which facilitate chol export (Tontonoz and Mangelsdorf 2003; Goldstein et al., 2006; Ikonen 2008; Goedeke and Fernandez-Hernando 2012; Luo et al., 2020). This section will give a simplified overview of the complex protein network that regulates these different stages of chol homeostasis (Figure 1A).

#### 2.1.1 *De novo* synthesis

In chol-poor conditions, nucleated cells activate the synthesis of new chol through the mevalonate pathway in the ER. In brief, two molecules of acetyl-coenzyme A (CoA) condense to form acetoacetyl-CoA which is added to a third acetyl-CoA molecule to produce one molecule of 3-hydroxy-3-methylglutaryl CoA (HMG-CoA) upon HMG-CoA synthase catalysis. Next, HMG-CoA is reduced to mevalonate by the integral ER membrane and rate-limiting HMG-CoA reductase (HMGCR). Then follows a succession of nearly 30 enzymatic reaction steps that convert mevalonate to squalene and afterwards to lanosterol and to chol (Ikonen 2008; Cerqueira et al., 2016; Shi et al., 2022). In the ER, chol can further be fatty acylated to form cholesteryl esters (CEs) or oxidized to form oxysterols. Additionally, chol can also be oxidized to bile acids and steroid hormones in hepatocytes and steroidogenic cells, respectively (Ikonen 2008). The chol biosynthetic pathway is tightly regulated by three key players, i.e. SREBP2, which regulates the transcription of genes encoding cholesterologenic enzymes, and HMGCR and squalene monooxygenase, two rate-limiting enzymes of the biosynthetic pathway [reviewed in (Brown and Goldstein 1997; Burg and Espenshade 2011; Chua et al., 2020; Luo et al., 2020)]. 

#### 2.1.2 Import

Besides the *de novo* synthesis, cells can acquire chol from the extracellular milieu through a receptor-mediated endocytic mechanism. Chol-carrying low-density lipoprotein (LDL) particles bind to LDL receptors (LDL-R) associated with clathrin-coated pits at the PM and are then delivered into early sorting endosomes. The LDL-R is recycled back to the cell surface while the chol-LDL complex is transported through compartments of the endocytic pathway where the low pH environment triggers hydrolysis of cholesteryl esters to provide free chol for cellular needs (Jeon and Blacklow 2005; Ikonen 2008; Goldstein and Brown 2009). The subsequent increase in intracellular chol generates a feedback regulation to stabilize the cell chol content (Goldstein and Brown 2009). Indeed, LDL-derived chol acts at different levels including suppression of the LDL-R gene transcription (Brown and Goldstein 1999), suppression of the HMGCR activity either by suppressing its gene transcription (Brown and Goldstein 1999) or accelerating the enzyme degradation (Gil et al., 1985) and activation of the chol-esterifying enzyme, acyl CoA: chol acyltransferase (ACAT) to store chol as CE-enriched droplets in the cytoplasm.

#### 2.1.3 Export

Excess chol is exported to the blood *via* ATP-binding cassette (ABC) subfamily A member 1 (ABCA1) or ABC subfamily G member 1 (ABCG1) to lipid-poor apolipoprotein A-I (ApoA-I), generating high-density lipoproteins (HDLs) (Gelissen et al., 2006; Rosenson et al., 2012; Daniil et al., 2013; Phillips 2014). Chol can also be exported to the intestinal lumen and bile ducts *via* ABCG5 and ABCG8 heterodimer (Luo et al., 2020). Nuclear receptor system LXRs are important regulators of chol export. In fact, high intracellular level of oxysterols activates the nuclear receptors of oxysterols LXRs which upregulates the transcription of ABCA1, allowing chol export (Ouvrier et al., 2009; Kuzu et al., 2016; Vona et al., 2021). On the other hand, expression of ABCA1 is downregulated by miR-33, which is co-transcribed with *SREBP* mRNAs during chol biosynthesis (Rayner et al., 2010).

#### 2.1.4 Esterification

A buffering mechanism takes places in normal cells to prevent free chol accumulation. Excess unesterified chol in the ER is transformed by integral membrane proteins ACAT into less toxic CEs to be stored in cytoplasmic lipid droplets (Chang et al., 2009; Luo et al., 2020). These lipid droplets are used for the production of plasma lipoproteins such as HDLs. The latter are then delivered from peripheral tissues, either to the liver and intestine for recycling or elimination, or to steroidogenic organs for steroid hormones production (Vona et al., 2021). Transcription of the two ACAT isozymes, ACAT1 and ACAT2, is not regulated by SREBPs or LXRs binding on their promoters but instead depends on various factors such as interferon-γ, all-trans-retinoic acid, synthetic glucocorticoid dexamethasone and tumor necrosis factor for ACAT1, as well as hepatocyte nuclear factors (HNF) 1α and 4α and homeobox protein CDX2 for ACAT2 (Chang et al., 2009; Luo et al., 2020).

### 2.2 Cholesterol physiological roles

Chol is involved in a large variety of physiological roles that can be classified into precursor functions and membrane-related effects.

#### 2.2.1 Precursor functions

Chol serves as a precursor for steroid hormones, oxysterols, vitamin D and bile acids (Vona et al., 2021) (Figure 1B). Steroid hormones are synthesized from a common precursor, pregnenolone, which is formed by enzymatic cleavage of a 6-carbon side-chain of the 27-carbon chol molecule (Hu et al., 2010). Steroid hormones are classified into five major groups: testosterone (androgen), estradiol (estrogen), progesterone (progestin), cortisol/corticosterone (glucocorticoid) and aldosterone (mineralocorticoids), all of which regulate physiological and pharmacological processes in the body (Falkenstein et al., 2000). Additionally, enzymatic and radical oxidation of chol produce oxygenated derivatives, termed oxysterols, which, as bioactive compounds, regulate chol homeostasis and mediate various degenerative and cancer-related disorders (Schroepfer 2000; Bielska et al., 2012; Griffiths and Wang 2019). Among the high diversity of oxysterols, one can cite the 1α,25-dihydroxyvitamin D_3_, the biologically active form of vitamin D_3_, and 7α-hydroxycholesterol, an intermediate in the classical bile acid synthesis pathway (Luu et al., 2016; Samadi et al., 2021).

#### 2.2.2 Membrane building block and key regulator of membrane properties

Chol is known to contribute to a large variety of membrane-related functions such as signaling events, cell:cell interactions and PM deformation processes including endocytosis and extracellular vesicle (EV) budding. This is made possible because chol is not only a major PM building block but it also regulates PM biophysical properties as well as lipid lateral and transversal distribution (Figure 1C). Indeed, chol is able to modify the PM rigidity by interacting with other membrane lipids (Simons and Vaz 2004; Gracià et al., 2010). Chol can increase the lateral ordering of membrane lipids by positioning in close proximity to the elongated and not very flexible saturated hydrocarbon chains of phospholipids compared with lipids that have unsaturated chains (Silvius 2003). Membranes with high chol levels also present increased thickness and are less permeable for drugs, often leading to the multidrug resistance featured in cancer cells (Alves et al., 2016; Preta 2020; Szlasa et al., 2020). More recently, chol has been proposed to be involved in the regulation of transversal lipid asymmetry (see Section 2.3). Chol is also heterogeneously distributed laterally in the membrane. Indeed, studies on bilayers with different lipid compositions have revealed that chol interacts with greater affinity with sphingomyelin (SM) > phosphatidylserine (PS) > phosphatidylcholine (PC) > phosphatidylethanolamine (PE) (van Dijck 1979; Silvius 2003). In cells, chol is not only enriched in chol-enriched domains (i.e., rafts, caveolae and stable submicrometric domains; see Section 2.3), but also appears crucial in the control of domain abundance and size [for a book chapter, see (Leonard et al., 2017a)].

### 2.3 Cholesterol membrane distribution

It is nowadays admitted that membranes are not homogenous, as illustrated by unequal lipid distribution among (i) different PMs, (ii) distinct intracellular compartments, (iii) inner vs*.* outer PM leaflets (i.e., transversal asymmetry), and (iv) the same PM leaflet (i.e., lateral heterogeneity). Those different levels of heterogeneity are particularly relevant for chol, as detailed here below (Figure 1D).

#### 2.3.1 Cell cholesterol content

The chol content is quite different from one PM to another. For example, whereas the PM chol content of human red blood cells (RBCs) and undifferentiated L6 myoblasts is comprised between 40 and 50%, it ranges between 30 and 40% for the Schwann cell line NF1T, CHO cells and human platelets and is around 10% in human alveolar macrophages and the fibroblast cell line NIH 3T3 (for a review, see (Carquin et al., 2016)). Since chol plays a dominant role in the regulation of membrane fluidity/rigidity, differential PM global and local chol levels will in turn differentially modulate membrane organization into domains and their related properties as well as the ability of cells to deform.

#### 2.3.2 Subcellular membrane cholesterol distribution

Besides strong cell-based differences, there is a considerable heterogeneity in membrane chol composition throughout different subcellular compartments. Thus, the molar ratio of chol to total phospholipids at steady-state in mammalian cells is ∼1.0 at the PM, ∼0.5 in late endosomes, ∼0.2 in the Golgi complex, ∼0.15 in the ER and ∼0.1 in the mitochondria (van Meer et al., 2008).

#### 2.3.3 Plasma membrane cholesterol transversal asymmetry

Although chol has been described as a highly mobile lipid that rapidly flip-flops across the membrane, favoring equal partition between both leaflets (Steck et al., 2002; Maxfield and van Meer 2010), other groups have instead suggested that its transbilayer distribution is not homogenous. Nevertheless, this is still debated and the mechanisms behind are still not well understood. In fact, the asymmetric transversal distribution could result from specific interactions with other components present in either leaflet. Thus, chol shows high affinity for sphingolipids and this association has been proposed as a mechanism behind sterol partition in the outer PM leaflet (Ramstedt and Slotte 2006; Menon 2018). However, sphingolipids may also exercise a different means of control by effectively pushing sterols into the inner leaflet, reducing sterol-sphingolipid interactions in the outer leaflet and affecting lipid packing in the inner leaflet (reviewed in (Menon 2018)). In addition, chol might be retained in the inner leaflet by interaction with specific lipids, as supported by the impairment of chol asymmetry in cells with low PS levels and its restoration upon exogeneous PS supply (for a review, see (Menon 2018)). It is nowadays proposed that the control of PM transversal chol asymmetry is coupled to the control of lateral distribution through ABCA1 (for a review, see (Wu et al., 2020)). Two series of arguments support the physiological relevance of chol transversal distribution. First, chol depletion leads to increased PS exposure at the surface of RBCs, suggesting a role of chol as phospholipid scrambling regulator (Arashiki et al., 2016). Second, the inner leaflet chol appears to be involved in diverse cellular processes such as modulation of cytoskeleton and motility and neurotransmitter receptor trafficking (Paukner et al., 2022).

#### 2.3.4 Plasma membrane cholesterol lateral asymmetry

Different types of lipid domains differing in size, stability, lipid content, regulation, dynamics and subcellular distribution are nowadays recognized. Those include lipid rafts, ceramide-enriched platforms, caveolae and stable submicrometric domains.

Lipid rafts are highly dynamic nanoscale (20–100 nm) assemblies enriched in chol and sphingolipids (Pike 2006) which contribute to the compartmentalization of signaling pathways by serving as platforms for the recruitment and concentration of membrane receptors, signaling molecules, kinases and phosphatases. In response to stimuli, lipid rafts change their size and composition and protect related proteins from degradation, thus promote the interaction between proteins and cell signal transduction. Moreover, rafts can protect signalling complexes from the effect of non-raft inhibitory proteins, allowing the isolation of proteins that can activate or inactivate certain pathways, facilitating or inhibiting downstream signal transduction (Mollinedo and Gajate 2020). Depending on the signal transduction needs, membrane proteins are transiently and reversibly recruited within lipid rafts *via* raft-targeting signals such as S-palmitoylation of transmembrane proteins or through a glycosylphosphatidylinositol (GPI) anchor (Sangiorgio et al., 2004; Chakrabandhu et al., 2007; Rossin et al., 2009; Babina et al., 2014). Lipid rafts also play other roles. For instance, their biophysical environment can induce conformational change of raft-resident proteins and thus modify their activity (Lingwood et al., 2011). Lipid rafts are also involved in immune signaling which depends on the phosphorylation state of immune receptors such as the T and B cell receptors and the high-affinity immunoglobulin E receptor (FcεRI), which are regulated by Src family kinases or phosphatases segregated in lipid rafts (Field et al., 1995; Gupta and DeFranco 2003; Dinic et al., 2015; Varshney et al., 2016). Furthermore, rafts are enriched in specific receptors for pathogens (e.g., CD4 for HIV) or toxins (*e.g*. glycosphingolipids for cholera toxin) (Teissier and Pecheur 2007; Iwabuchi 2015). Finally, proteins associated with malignancy are known to be recruited in rafts which, in turn, regulate cancer cell signaling pathways such as tumor cell growth, adhesion, migration, invasion, survival and apoptosis (Sezgin et al., 2017; Vona et al., 2021). This is the topic of Section 4.

Many receptors or stress stimuli have been shown to transform small rafts into ceramide-enriched domains through the activation and translocation of the acid sphingomyelinase to the outer PM leaflet. These platforms cluster receptors, recruit intracellular signaling molecules and appear to exclude inhibitory signaling factors. Through this mechanism, ceramide-enriched platforms contribute to apoptosis by clustering the death receptor CD95 (APO-1/Fas) and ultimately forming the death-inducing signaling complex (DISC) [see (Gulbins and Li, 2006) for a review and section 4.3]. It should be noticed that the formation of ceramide-enriched domains could also occur without the presence of rafts.

Caveolae are small PM pits of 60–80 nm in size. They represent a characteristic structure of many vertebrate cells but are predominantly found in cardiac muscle, endothelial cells and adipocytes (Parton et al., 2020; Low and Laiho 2022). Caveolae are considered as specialized lipid domains. Indeed, like rafts, they are enriched in chol but, in contrast to rafts, their formation, dynamics and pathophysiology depend on two classes of proteins, the membrane caveolins and the cytoplasmic cavins. Caveolin-1 (Cav-1) is a chol-binding protein with a chol recognition/interaction amino acid consensus motif that significantly enriches chol within the caveolar domain (Murata et al., 1995; Epand et al., 2005). It has been estimated that one caveolae can contain up to 22,000 chol molecules (Ortegren et al., 2004). Moreover, chol distribution within the Golgi and the PM depends on Cav-1 and/or caveolae expression (Pol et al., 2005; Hayer et al., 2010), suggesting that chol homeostasis is linked to Cav-1 and caveolae. Besides chol, other lipids are found to be enriched in caveolae, including SM and glycosphingolipids at the outer PM leaflet and PS and phosphatidylinositol-4,5-bisphosphate (PIP2) at the inner one (Ortegren et al., 2004; Fujita et al., 2009; Fairn et al., 2011). However, the precise composition is still not fully understood (Parton et al., 2020). Caveolae have been suggested to contribute to a wide range of cellular processes including lipid regulation, signaling events, endocytosis, transcytosis, cell adhesion, migration and mechanoprotection (reviewed in (Parton et al., 2020)).

More recently, stable submicrometric lipid domains have been reported in a variety of cells thanks to the development of new probes and innovative imaging methods (Carquin et al., 2014; D'Auria et al., 2013; Sanchez et al., 2012; Carquin et al., 2015; Tyteca et al., 2010; Bach and Bramkamp 2013; Grossmann et al., 2007; Carquin et al., 2016). Nevertheless, data had to be interpreted with caution due to potential false interpretations of lipid domains resulting from surface vesicular structures and/or membrane protrusions. To circumvent this difficulty, our group focused on RBCs as the simplest and best characterized eukaryotic cell system presenting a flat surface without membrane protrusions and which do not make endocytosis. We revealed the coexistence of three populations of submicrometric domains which exhibit differential abundance, lipid enrichment, RBC curvature association and roles: chol-, GM1 ganglioside/PC/chol- and SM/PC/chol-enriched domains (Conrard et al., 2018; Carquin et al., 2014; D'Auria et al., 2013; D'Auria et al., 2011; Tyteca et al., 2010; Carquin et al., 2015; Leonard et al., 2018; Leonard et al., 2017a). Although lipid domains have been well studied in RBCs, data on their existence, biogenesis and physiological roles in nucleated cells are sparse. For more details on lipid domains biogenesis and roles, see (Carquin et al., 2016). For alteration of lipid domains in cancer, see next section.

## 3 Alteration of membrane cholesterol content and distribution in cancer

Although chol-enriched domains are nowadays recognized as contributing to cancer cell proliferation, survival, death and invasion with important implications in tumor progression (see Section 4), whether and how those domains are modified in cancer remains poorly understood. Another concern is whether and how the PM transversal distribution of chol is impaired in malignant cells and whether it is coupled to chol lateral heterogeneity alteration. These are key questions that need to be answered before we move forward implementing a lipid domain-mediated approach as anticancer therapy **(**see Section 5). Therefore, Section 3 aims to summarize the few sometimes contradictory literature data on membrane chol composition and biophysical properties (Section 3.1), chol-enriched domain abundance and properties (Sections 3.2–3.4) and chol transversal asymmetry (Section 3.5) in cancer cells.

### 3.1 Cholesterol content and membrane biophysical properties

**FIGURE 2:**
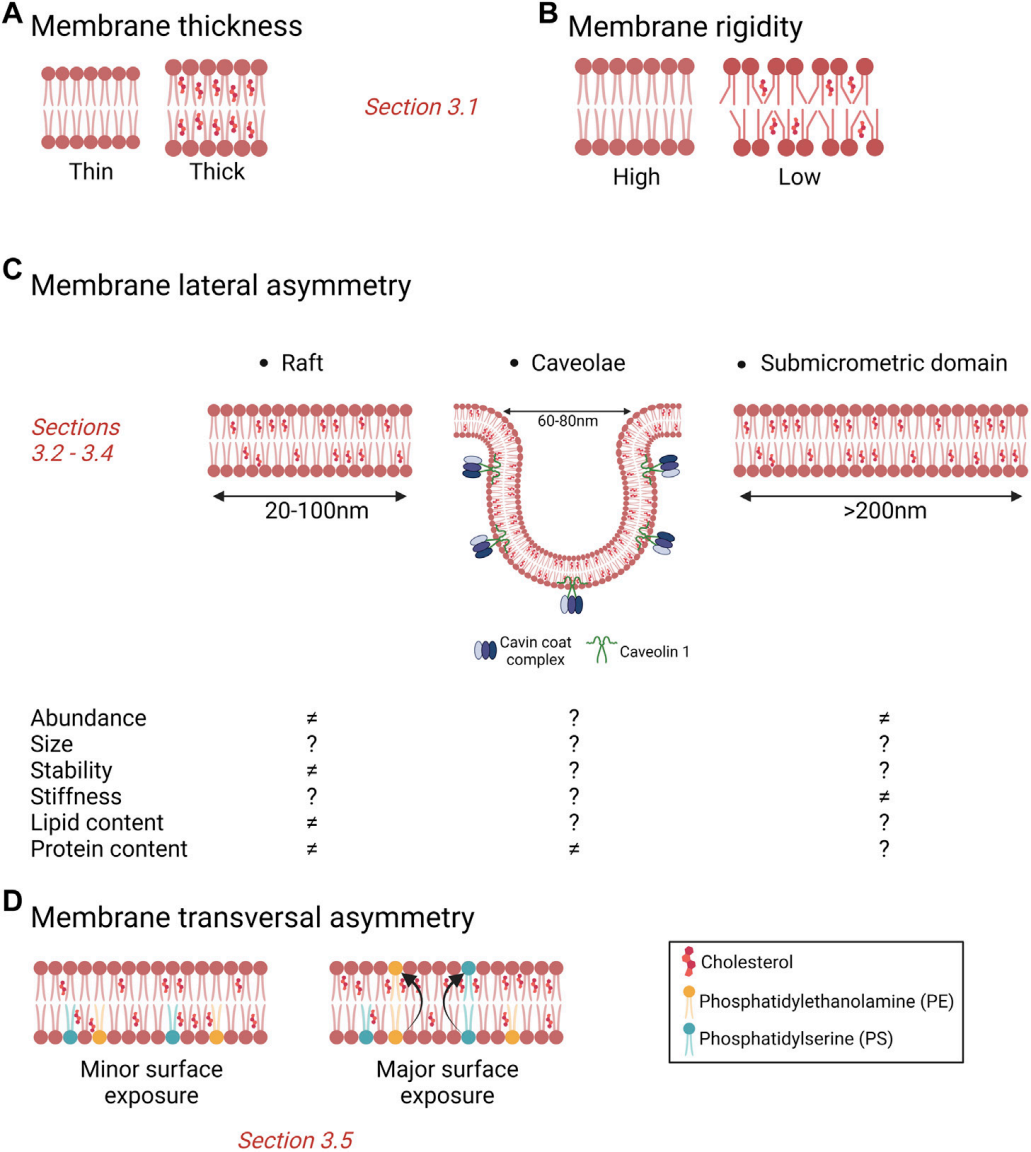
Importance of cholesterol for membrane biophysical properties and heterogeneity. **(A,B)** Membrane chol content modulates membrane thickness (A) and rigidity (B), two membrane biophysical properties. **(C)** Membrane chol is a key component of lipid rafts, caveolae and submicrometric domains. Those different types of domains can be modified in abundance, size, stability, stiffness and contents in cancer cells but data are sparse and effects sometimes divergent. ≠, alteration vs. non-tumorigenic cells; ?, unknown. **(D)** Surface chol and PS contents have been proposed to be increased in cancer cells as compared to non-tumorigenic cells. See the Section 3 of the text for further details.

Chol content is generally described as altered in cancer, with consequences in terms of membrane biophysical properties including thickness and rigidity (Figures 2A,B). However, conflicting data arise even for a same type of cancer. This is particularly true for breast cancer. On one hand, higher expression of chol biosynthesis genes associates with worse prognosis of basal-like breast cancer patients (Ehmsen et al., 2019). Also, multidrug resistant cells exhibit higher chol that turns the PM thicker and more rigid and thus less permeable for drugs (for a review, (Peetla et al., 2013)). On the other hand, lower chol levels in metastatic cells correlate with a more deformable PM, increasing its invading capacity (Sok et al., 2002; Zeisig et al., 2007). Likewise, lower chol levels in breast cancer cell lines lead to increased membrane fluidity, promoting migration and invasion (Yang et al., 2018; Zhang et al., 2019; Zhao et al., 2019). In tumor tissues of thyroid, uterine, ovarian and renal cancers increased chol contents as compared to normal tissues have been shown (Kim et al., 2018; Mayengbam et al., 2021).

Thus, the dysregulation of membrane chol content and related biophysical properties in cancer is the object of debate, which could be explained by the following non-mutually exclusive hypotheses. First, it could result from the comparison of non-isogenic cell models as well as the use of different approaches to evaluate membrane rigidity without any distinction between the membrane and the cytoskeleton contributions. This consideration is well illustrated by our recent study using three mammary cell lines with increasing invasion potential but the same genetic background (i.e., non-tumorigenic MCF10A, pre-malignant MCF10AT and malignant MCF10CAIa cells). Thus, thanks to atomic force microscopy at different modes, we revealed the specific stiffening of the PM of the malignant cell line that is uncoupled from its elastic cytoskeletal properties and this despite similar total chol content in the three cell lines (Dumitru et al., 2020; Maja et al., 2022). Second, as explained in Section 2.3, various cell types exhibit different membrane chol levels, a feature also relevant to cancer cells. Consequently, if the comparison is made with a non-isogenic cell line, erroneous conclusions can be drawn regarding the alteration of chol content in cancer. Third, changes can depend on the type or even the sub-type of cancers. Fourth, chol could have opposite roles depending on cancer progression. In line with this idea, cancer cells undergoing metastasis have been proposed to exhibit lower membrane chol levels to favor membrane fluidity (Zhao et al., 2019; Szlasa et al., 2020). Fifth, chol lateral and transversal heterogeneity have often been neglected and should be considered as they can be affected in cancer, as discussed in Sections 3.2–3.5.

### 3.2 Lipid rafts

Based on the assumption that cancer cells generally present higher chol contents (see Section 3.1), they are usually associated with elevated levels of lipid rafts (Figure 2C, left). However, the link between total chol content and its distribution in domains could not be so evident. Indeed, by directly comparing cell lines with the same genetic background, we showed that, despite a similar chol content in malignant vs*.* non-malignant cells, malignant cells specifically exhibit a ∼50% increase of chol at their external PM leaflet and its clustering in submicrometric domains (Maja et al., 2022) (see Section 3.4). Nevertheless, some microscopy and flow cytometry studies support the above suggestion of elevated rafts in cancer cells. Thus, a number of human prostate and breast cancer cell lines show stronger chol and GM1 staining as compared with their non-tumorigenic cell line counterparts (Li et al., 2006). Likewise, a higher chol and increased raft levels have been reported in tumorigenic vs*.* non-tumorigenic melanoma cells by flow cytometry analysis upon labeling with filipin and di-4-ANEPPDHQ (Levin-Gromiko et al., 2014). The lack of data on lipid raft abundance and size could result from limited availability of appropriate lipid tools as well as efficient imaging techniques compatible with both live cell microscopy and lipid raft size and transient properties.

There is not much more data on lipid raft properties in cancer. Through an integrated analysis of lipid raft proteomics data sets modeling progression in breast cancer, melanoma and renal cell carcinoma, Shah and others have revealed the increased cytoskeleton-mediated stabilization of lipid rafts with greater molecular interactions as a common, functional and reversible feature of cancer cells (Shah et al., 2016).

One of the least well-resolved questions in the field is whether the lipid and protein composition of rafts is altered in cancer cells. Many studies converge in this direction but they are essentially based on indirect evidence related to the overall cell lipid content and the association/dissociation of proteins in the rafts, combined or not with the alteration of the membrane chol content. For example, in diffuse B cell lymphoma, the Apoptotic peptidase activating factor 1 (Apaf-1) involved in the apoptosome is abnormally distributed in rafts instead of the cytosol. Chol depletion by methyl-β-cyclodextrin (mβCD) allows the restoration of Apaf-1 cytosol localization and the correct apoptosome assembly (Hirpara et al., 2016). In colon cancer cells, the carcinoembryogenic antigen (CEA) is abnormally localized at the basolateral side, which can result from impaired GPI anchorage of CEA in rafts due to the increased pH within the Golgi (Kokkonen et al., 2018). In breast cancer cells, whereas CD44 and ezrin localize in different membrane regions at resting state, CD44 association with rafts decreases and its interaction with ezrin increases after induction of migration (Donatello et al., 2012). As another example related to breast cancer cells, the enrichment of rafts in n-3 polyunsaturated fatty acids (PUFAs) has been linked to modifications of cell signaling processes leading to cell death (Schley et al., 2007).

As mentioned in Section 2.3, many receptors or stress stimuli have been shown to transform rafts into ceramide-enriched platforms which may be crucial for numerous functions including the induction of apoptosis (for a review, see (Gulbins and Li 2006)). A question that arises is whether those ceramide-enriched platforms present differential abundance or properties in cancer cells, with potential consequences in the decision to apoptosis vs*.* cell survival and proliferation. In line with this hypothesis, low levels of surface membrane-associated neutral sphingomyelinase 2, responsible for SM hydrolysis into ceramide, are associated with early recurrence of hepatic cell carcinoma after surgery (Revill et al., 2013). Moreover, several cancer cells show significant alterations in the enzymes involved in ceramide metabolism, often resulting in loss of ceramide (Canals and Hannun 2013) and contributing to consider ceramide as a tumor suppressor lipid. Alterations of SM/ceramide pathway influence the cell death process and the survival of cancer cells after ionizing radiation (Aureli et al., 2014). In addition, a functional role for ceramide kinase in breast cancer recurrence has been identified (Payne et al., 2014). For reviews dedicated to the roles of sphingolipids in cancer, we refer the reader to (Codini et al., 2021; Tallima et al., 2021).

### 3.3 Caveolae

Epidemiological, molecular and clinical studies converge to the conclusion that caveolae are involved in tumor progression and cancer treatment resistance. This conclusion is mainly based on alterations of caveolae-associated molecules (Figure 2C, middle), summarized here below.

First, the expression of Cav-1 is modulated in tumors and in tumor stroma and those modulations are connected with progression of several cancers (Lamaze and Torrino 2015; Low and Laiho 2022). The mechanisms by which Cav-1 contributes to the progression of breast cancer are discussed in (Qian et al., 2019). Nevertheless, different studies have produced opposite data, Cav-1 being considered either as a tumor suppressor or an oncogene (Lamaze and Torrino 2015). In an attempt to reconcile the different observations, Lamaze and Torrino have proposed that a biphasic expression pattern could be correlated with distinct Cav-1 functions (Lamaze and Torrino 2015).

Second, the expression of cavins is also altered in several cancer types. Thus, Cavin1 has been shown to be downregulated in prostate, lung and breast cancers while Cavin2 is downregulated in breast, kidney and prostate tumors. Cavin3 is frequently inactivated in ovarian cancers and downregulated in breast cancer cell lines and breast tumor tissues. For a review on cavin expression and function in cancer, we refer the reader to (Lamaze and Torrino 2015) and (Low and Laiho 2022).

Third, alterations of caveolin and cavin expression and function are connected with cancer treatment resistance. This topic has been recently reviewed by (Low and Laiho 2022) in different types of cancer and by (Qian et al., 2019) in breast cancer. Based on the facts that endothelial cells are critical determinants of the radiation response of tumors (Klein et al., 2015; Klein 2018) and that Cav-1 deficient tumor cells are more sensitive to radiation therapy (Klein et al., 2015), Ketteler and others recently explored the potential link between the Cav-1-dependent radiation response of endothelial cells and signaling mediated by ceramide-enriched platforms. They found that Cav-1 regulates the ceramide-dependent PM reorganization which in turn affects the radiation response of endothelial cells and adjacent prostate cancer cells (Ketteler et al., 2020).

Thus, alterations of caveolae-associated molecules are documented. In contrast, whether caveolae themselves are altered in structure, abundance, lipid composition and function remains poorly understood.

### 3.4 Stable submicrometric domains

**FIGURE 3:**
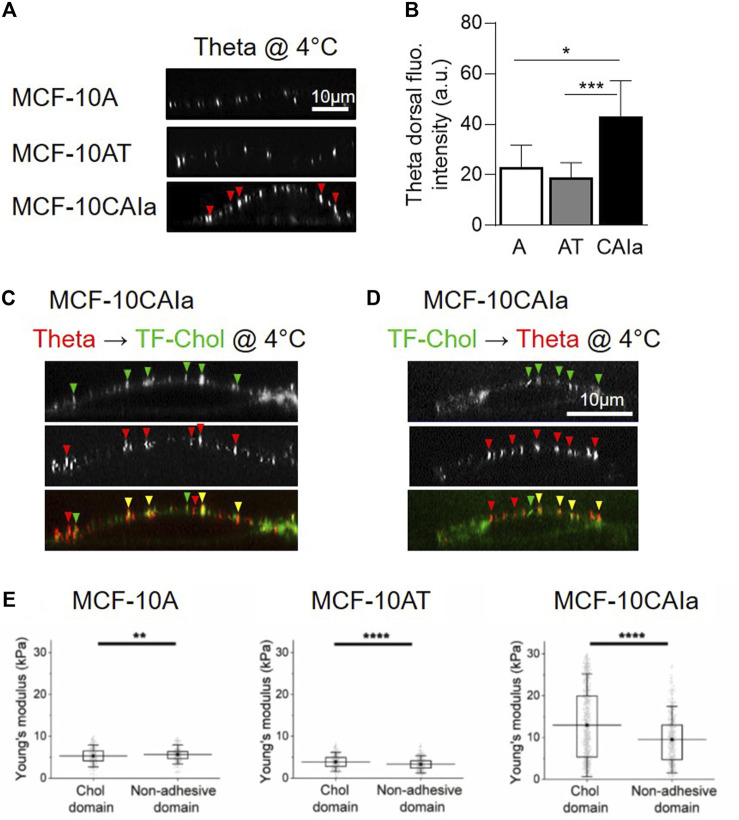
Specific increase of surface cholesterol content, submicrometric cholesterol-enriched domain abundance and membrane stiffness on malignant breast cancer cells. **(A,B)** Specific increase of dorsal chol and distribution in submicrometric domains (arrowheads) at the surface of the malignant MCF-10CAIa cells. X–Z reconstructions of confocal images of normal MCF-10A, pre-malignant MCF-10AT and malignant MCF-10CAIa cell lines plated on glass coverslips, labeled at 4 °C with the mCherry-Theta toxin fragment specific to endogenous chol (A) and quantified for the Theta dorsal fluorescence (fluo.) intensity (B). **(C,D)** Evidence for submicrometric chol-enriched domains at the dorsal face of malignant MCF-10CAIa cells by two complementary chol-specific probes, i.e., endogenous chol decoration by mCherry-Theta toxin fragment (Theta) (as in A,B) and PM insertion of TopFluor-Chol (TF-Chol). Yellow arrowheads, colocalization. **(E)** Specific stiffening of chol-enriched domains with respect to non-adhesive areas on malignant MCF-10CAIa cells. **(A–D)** Adapted from Maja et al., 2022; **(E)** Adapted from Dumitru et al., 2020. See the Section 3.4 of the text for further details.

As highlighted in Section 2.3, the development of new probes and imaging methods during the past decades has allowed to evidence submicrometric domains in a variety of living cells. A key question is whether those domains could be altered in cancer cells. We have recently addressed this issue by directly comparing the three above-mentioned mammary cell lines with increased invasive potential but the same genetic background (i.e., the MCF10A cell line series), for chol membrane distribution using three complementary approaches: (i) atomic force microscopy with Theta toxin fragment-derivatized tips to analyze chol distribution in a label-free manner with high spatial resolution; (ii) direct cell labeling at 4 °C with the fluorescent Theta toxin fragment; and (iii) PM insertion of the fluorescent chol analog TopFluor-Chol. We revealed that malignant cell lines exhibit higher surface chol content as compared to non-tumorigenic and pre-malignant cell lines and that this surface chol clusters into abundant chol-enriched submicrometric domains (Figure 2C, right; Figures 3A–D). Those features have been similarly observed in the highly invasive MDA-MB-231 breast cancer cell line which exhibits the common TP53 mutation (Dumitru et al., 2020; Maja et al., 2022).

Importantly, surface chol-enriched domains in malignant cells present a remarkable specific stiffening as compared to bulk membrane areas (Dumitru et al., 2020) (Figure 3E). The fact that this stiffening has been specifically observed in malignant cells leads us to suggest that the local composition and/or cytoskeleton anchoring of chol-enriched domains could be different in malignant cells than in pre-malignant and non-tumorigenic cells, but this remains to be demonstrated.

Our data also suggest that those chol-enriched domains coexist with another type of submicrometric domains, also enriched in chol but showing differential sensitivity to chol depletion, ability to be internalized and actin cytoskeleton-dependence (Maja et al., 2022). However, the sphingolipid and phospholipid composition of this second type of domains remains to be determined. Moreover, it will be important to evaluate upon cancer progression the relative proportion of these two types of submicrometric domains as well as their potential coexistence with lipid rafts and ceramide-enriched platforms.

### 3.5 Cholesterol transversal distribution

Only a few studies have been dedicated to the transversal distribution of chol in tumor cells. We recently showed a higher surface chol content in malignant cell lines as compared to non-tumorigenic and pre-malignant mammary cell lines (Dumitru et al., 2020; Maja et al., 2022). Although the mechanistics behind this increased chol surface exposure is not understood, literature data suggest that the different mechanisms proposed to support PM chol transversal distribution in normal cells (see Section 2.3) are altered in cancer. First, both sphingolipid levels and functions are impaired in cancer (for reviews, see (Codini et al., 2021; Tallima et al., 2021)). Second, although cell surface level of PS is classically thought to be exclusively found in apoptotic cells, viable cancer cells also present elevated levels of PS on their outer PM leaflet (Blanco et al., 2014; Vallabhapurapu et al., 2015; Birge et al., 2016) (Figure 2D). Third, several studies indicate that the chol transporter ABCA1 is involved in several types of cancer cells but contradictory data arise. On one hand, decreased expression of ABCA1 has been observed in (i) cancer vs*.* normal human breast tissues (Schimanski et al., 2010); and (ii) liver tissues from patients with hepatocellular carcinoma vs*.* peripheral blood leukocytes (Moustafa et al., 2004). On the other hand, in triple-negative breast cancer tissues, the expression of ABCA1 is higher than in non-cancerous mammary tissues (Pan et al., 2019). Likewise, in epithelial ovarian cancer, elevated ABCA1 expression associates with poor clinical outcome and ABCA1 suppression significantly reduces the growth, motility and colony formation of epithelial ovarian cancer cell lines and the size of epithelial ovarian cancer spheroids (Gao et al., 2022). Interestingly, perturbing chol efflux through suppression of ABCA1 leads to malignant characteristics impairment (Zhao et al., 2016; Torres-Adorno et al., 2019).

## 4 Contribution of cholesterol-enriched domains in cancer

Although the involvement of chol in tumor processes such as survival, proliferation and migration has been widely reported, its role in cancer development is still controversial (Vona et al., 2021). Moreover, most studies have been dedicated to the role of cellular chol and not of membrane chol specifically. In this section, we focus on studies describing the contribution of chol-enriched domains (mainly lipid rafts and submicrometric domains) in cancer cell survival and proliferation (Section 4.1), adhesion, migration and invasion (Section 4.2), apoptosis (Section 4.3) as well as interaction with the microenvironment (Section 4.4) and release of EVs (Section 4.5).

### 4.1 Cell survival and proliferation

One of the hallmarks of cancer is the sustained survival and proliferative signaling of tumor cells. Fast dividing cancer cells require membrane formation and thus an increased supply of chol. Many preclinical studies have suggested a link between tumor growth and altered chol metabolism and exogenous supply (Silvente-Poirot and Poirot 2012; Huang et al., 2020; Xu et al., 2020; Raftopulos et al., 2022). Moreover, a wide number of cell survival and proliferation proteins can be found in lipid rafts, as discussed below.

#### 4.1.1 PI3K/Akt pathway

The PI3K/Akt pathway is a major cell survival pathway (Payrastre and Cocco 2015). In response to growth factors such as insulin-like growth factor (IGF)-1 (see Section 4.1.2), the heterodimeric enzyme phosphatidylinositol 3-kinase (PI3K) is recruited by the insulin receptor substrates (IRS)-1/2 and phosphorylates PIP2 to produce phosphatidylinositol-3,4,5-trisphosphate (PIP3) in the PM. PIP3 then binds with high affinity to the pleckstrin homology (PH) domain of the effector kinase Akt. PIP3 also promotes membrane recruitment of PI-dependent protein kinase 1 (PDK1) and mammalian target of rapamycin complex 2 (mTORC2) that both phosphorylate and activate Akt, that in turn regulates numerous target proteins involved in cell proliferation, survival and growth (Manning and Toker 2017; Shi et al., 2019; Jiang et al., 2020; Rascio et al., 2021). Due to its overexpression and activation in many solid and hematological tumors, Akt is considered as a target in cancer therapy (Bellacosa et al., 2005; Song et al., 2019).

The spatial compartmentalization of PI3K/Akt signaling in lipid rafts is essential to recruit and activate Akt and to trigger the pathway (Lasserre et al., 2008; Gao et al., 2011). It has indeed been shown in the radiation-resistant triple negative breast cancer cell line (MDA-MB-231-IR) that membrane binding of the natural phenolic lipid 10-gingerol modulates lipid domain, affecting the PI3K/Akt signaling pathway and thereby inhibiting cancer cell proliferation, migration and invasion while inducing apoptosis (Ediriweera et al., 2020). Another study has shown that displacing PI3K, Akt, PDK1 and mTOR from lipid rafts with the raft-targeted antitumor ether lipid edelfosine leads to Akt dephosphorylation and apoptosis (Reis-Sobreiro et al., 2013).

**FIGURE 4:**
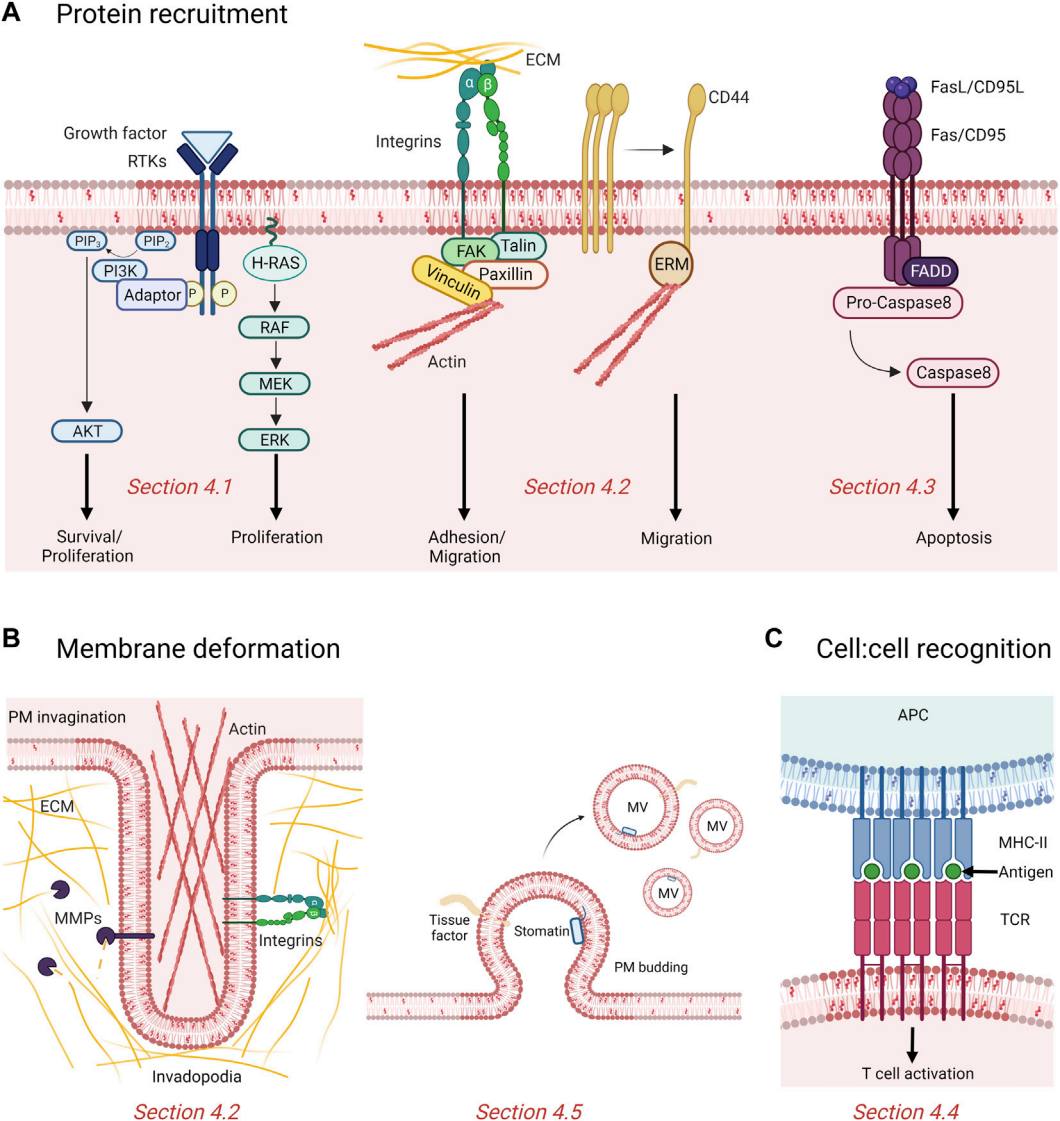
Contribution of rafts and submicrometric cholesterol-enriched domains in cancer. **(A)** Rafts recruit and cluster proteins involved in proliferation, survival, adhesion, migration and apoptosis. Binding of growth factors induce phosphorylation (P) and activation of tyrosine kinase receptors (RTKs) recruited in raft, which then activate downstream signaling cascades like the phosphatidylinositol 3-kinase (PI3K)/AKT and MAPK pathway promoting cell proliferation and survival. Domains also promote adhesion and migration of cancer cells by recruitment of integrins and displacement of the cell surface adhesion receptor CD44 to non-raft membrane areas. Fas/CD95 death receptors cluster in rafts to recruit the adaptor molecule Fas-associated death domain (FADD) which activates the caspase-mediated pro-apoptotic signaling pathway. PIP2, phosphatidylinositol (4,5)-bisphosphate; PIP3, phosphatidylinositol (3,4,5)-trisphosphate; ECM, extracellular matrix; ERM, Ezrin/Radixin/Moesin; FAK, focal adhesion kinase. **(B)** Chol-enriched domains participate in membrane deformation either by forming actin-based and proteolytically-active invadopodia or releasing microvesicles (MVs). MMPs, matrix metalloproteinases. **(C)** Rafts mediate recognition between cells. The antigen presented at the surface of the antigen presenting cell (APC) by the major histocompatibility complex (MHC) class II is recognized by the T cell receptors (TCR) clustered in rafts and induce T cell activation. See the Section 4 of the text for further details.

Moreover, several studies indicate that the PM chol content is required to regulate the PI3K/Akt pathway. Thus, inhibition of chol synthesis by simvastatin affects Akt-mediated survival of prostate cancer cells and xenografts (Zhuang et al., 2005). Activation of the PI3K/Akt pathway is impaired to the benefit of apoptosis upon membrane chol targeting by mβCD, as evidenced in macrophages, lung adenocarcinoma and leukemia cell lines (Motoyama et al., 2009). Depletion of membrane chol content blocks Akt binding to PDK1 at the PM, increasing the sensitivity of three isogenic epidermal human keratinocyte cell lines to apoptosis stimuli (Calay et al., 2010) (Figure 4A, left).

#### 4.1.2 IGFR pathway

In the IGF system, binding of IGF-1 and IGF-2 to their raft-localized tyrosine kinase receptors induces receptor phosphorylation. The activated receptors recruit and phosphorylate intracellular adaptor proteins such as the IRS-1 and IRS-2, which then active downstream signaling cascades like the PI3K/Akt and the MAPK pathways, promoting cell proliferation and survival (Pollak 2012; Mollinedo and Gajate 2020; Li et al., 2022). In cancer, the IGF pathway has been reported to be overexpressed and overactivated which is crucial for tumor development (Mollinedo and Gajate, 2015; Mollinedo and Gajate, 2020).

As the IGF system and Akt pathway occur in lipid rafts, they highly depend on membrane chol to mediate proliferation and survival of cancer cells. Indeed, chol depletion by mβCD and inhibition of chol biosynthesis with 25-hydroxycholesterol inhibit IGF-1-mediated phosphorylation of Akt and cell survival (Romanelli et al., 2009). Disruption of lipid rafts by mβCD also impairs the activation of the PI3K/Akt pathway in human lung adenocarcinoma A549 cells and Jurkat cells (Motoyama et al., 2009) (Figure 4A, left).

#### 4.1.3 Ras signaling

The Ras small GTPase family is involved in a variety of signaling processes such as cell growth, proliferation, survival, differentiation, adhesion and motility (Murugan et al., 2019). In human, this family is composed of more than 39 proteins, the most characterized being H-Ras, K-Ras and N-Ras. Activated Ras recruits various signaling molecules such as the PI3K that initiates Akt/mTOR-mediated cell growth (Nussinov et al., 2014), and serine/threonine kinases of the Raf family that activate the ERK-MAP kinase pathway leading to cell proliferation (Simons and Toomre, 2000) (Figure 4A, left).

While both K-Ras and H-Ras are localized in the PM, only H-Ras partitions into lipid rafts due to its palmitoylation and is thus dependent on appropriate membrane chol level to mediate its activity (Hancock et al., 1990; Roy et al., 1999). In fact, Roy and coll. have observed that chol depletion by mβCD specifically and reversibly inhibits H-Ras-mediated activation of Raf but not that mediated by K-Ras, and that this effect mimics the one observed by expressing a dominant-negative mutant of caveolin (Roy et al., 1999). Interestingly, experiments showed that GDP-bound H-Ras depends on PM chol to segregate into nanoclusters while GTP-bound H-Ras forms chol-independent clusters, strongly suggesting that PM chol is crucial for appropriate Ras signaling (Prior et al., 2003).

#### 4.1.4 EGFR pathway

EGFR family has been widely studied in the context of cancer due to its involvement in cell proliferation and its upregulation in human tumors such as breast, ovary, lung, colorectal, pancreas, head and neck, bladder and kidney and glioblastoma (Mitsudomi and Yatabe 2010). Binding of EGF to the EGFR tyrosine kinase induces receptor dimerization, trans-autophosphorylation and the recruitment of signaling proteins and adaptors through their own phosphotyrosine-binding SH2 domains (Wee and Wang 2017). These downstream signaling proteins then initiate diverse signal transduction cascades such as the MAPK, Akt and JNK pathways, leading to the production of CYCLIN D1 which initiates G1/S cell cycle progression and ultimately promoting DNA synthesis and cell proliferation (Oda et al., 2005; Wee and Wang 2017).

EGFR is localized in lipid rafts (Pike et al., 2005), suggesting a lipid-based signaling. Accordingly, while studies reported that the GM3 ganglioside strongly inhibits auto-phosphorylation and tyrosine kinase activity of EGFR in cells and proteoliposomes (Bremer et al., 1986; Zhou et al., 1994; Coskun et al., 2011), depletion of PM chol on the other hand appears to activate the receptor in a ligand-independent manner (Chen and Resh 2002; Pike and Casey 2002; Saffarian et al., 2007). Conversely, PM chol lowering agents have been shown to impair EGFR pathway, thereby altering survival of prostate cancer cells and xenografts (Zhuang et al., 2005; Oh et al., 2007). Although the mitogenic response to EGF is impaired in mβCD-treated keratinocytes, chol depletion still stimulates cell growth *via* EGFR, possibly through the formation of micrometric clusters containing activated and phosphorylated form of EGFR (Lambert et al., 2006). Otherwise, localization of EGFR in lipid rafts correlates with EGFR tyrosine kinase inhibitor resistance of breast cancer cell lines which can still mediate Akt signaling in the absence of EGFR kinase activity. However, upon chol depletion using lovastatin, the resistance of cancer cell lines to the EGFR tyrosine kinase inhibitor gefitinib decreases (Irwin et al., 2011). Lovastatin also modulates PM rigidity and fluidity, promoting endocytic degradation of the tyrosine kinase receptor ErbB2 and thus synergizing with the tyrosine kinase inhibitor lapatinib to impair ErbB2-positive breast cancer growth (Zhang et al., 2019). Moreover, Chen and coll. confirmed the role of membrane chol in the resistance of non-small cell lung cancer cells to tyrosine kinase inhibitor as chol depletion reversibly enhances the gefitinib inhibition of EGFR, Akt-1, MEK1/2, and ERK1/2 phosphorylation (Chen et al., 2018). Altogether, targeting PM chol along with EGFR tyrosine kinase inhibitor treatment could improve actual therapeutic strategy to bypass gefitinib resistance.

#### 4.1.5 Studies integration

Taken together, above studies point out the sensitive equilibrium between survival and death pathways through Akt signaling as well as the importance of membrane chol and lipid rafts in the regulation of cell survival and proliferation signaling in cancer. Nevertheless, the real role of rafts in proliferation and survival remains to be clearly established as chol-depleting agents are not specific to lipid rafts, disturbing above all the global chol content and being able to also modulate the cytoskeleton organization and sometimes even causing non-specific or toxic effects in case of excessive depletion.

### 4.2 Cell adhesion, migration and invasion

Cell adhesion, migration and invasion are other fundamental processes of cancer and metastasis. To expand from a primary tumor into nearby environment, cancer cells must adequately respond to mechanical stresses while navigating through diverse microenvironments. In this regard, PM chol mediates a series of events at both cellular and molecular levels, such as modulation of PM nanomechanical properties, cell morphology, adhesion to the surrounding substrate and degradation of the extracellular matrix (ECM)-mediated by matrix metalloproteinases (MMPs).

#### 4.2.1 Cholesterol global content

At the cellular level, many studies have established a link between PM chol content and migration and invasion capacities, but data are often contradictory. On one hand, lower chol levels lead to increased membrane fluidity, which promote migration and invasion of human liver and breast cancer cells (Yang et al., 2018; Zhang et al., 2019; Zhao et al., 2019). Additionally, lung and breast metastatic cells exhibit lower chol levels that correlate with a more deformable PM and increased invading capacity compared to non-invading cells (Sok et al., 2002; Zeisig et al., 2007). On the other hand, chol supplementation favors migration and invasion of renal carcinoma cells (Liu et al., 2018) while depletion of membrane chol impairs breast tumor cells migration (Guerra et al., 2016; Kumar et al., 2018). Conflicting data could arise from the different alterations of chol metabolism that appear to be tumor- and subtype-specific, the lack of isogenic models to directly compare cancer cells to their healthy counterparts as well as the differential residual chol content after modulation.

#### 4.2.2 Cholesterol surface exposure

By directly visualizing the surface chol by high-resolution vital imaging upon cell labeling at 4 °C with complementary validated probes, we revealed a higher chol content at the surface of malignant MCF10CAIa and MDA-MB-231 cell lines (Figures 3A–D). In addition, this higher chol content correlates with PM stiffening and promotes cell invasion (Maja et al., 2022; Dumitru et al, 2020).

#### 4.2.3 Cholesterol-enriched domains and cancer cell adhesion and migration

At the molecular level, many studies reported the association of cell adhesion molecules with lipid rafts (Figure 4A, middle).

Among those adhesion molecules, the cell surface adhesion receptor CD44 has been widely described in the context of tumor cell signaling, adhesion and migration (Senbanjo and Chellaiah 2017; Mollinedo and Gajate 2020; Vona et al., 2021). Displacement of CD44 from lipid rafts to non-raft membrane regions allows for CD44 shedding by the metallopeptidase ADAM10 which induces human glioblastoma cell migration (Murai et al., 2004; Murai 2012). Additionally, localization of CD44 within lipid rafts decreases during migration of highly invasive MDA-MB-231 breast cancer cells (Donatello et al., 2012; Babina et al., 2014). Interestingly, many studies reported the importance of adequate levels of PM chol for CD44-mediated cancer cell migration. Thus, lipid raft disruption by mβCD enhances CD44 shedding and suppresses glioma and pancreatic cancer cell migration (Murai et al., 2011). Similar effects were observed after disorganizing lipid rafts with simvastatin and filipin (Murai et al., 2011; Murai, 2012; Murai, 2015). In hepatocellular carcinoma, high levels of chol promote CD44 translocation in lipid rafts, abolishing its interaction with the actin-binding protein Ezrin outside rafts and leading to decreased cell migration (Yang et al., 2018).

While particular attention has been paid to the highly expressed CD44, several studies also showed that other membrane proteins such as the amyloid precursor protein (APP; (Kojro et al., 2001)), the interleukin-6 receptor (IL-6R; (Matthews et al., 2003)), the lymphoid activation marker CD30 (von Tresckow et al., 2004) and the lipoprotein receptor-related protein-1 (LRP-1; (Selvais et al., 2011)) also undergo shedding by ADAM family proteinases upon chol depletion. In breast carcinoma, cleavage of the L1-cell adhesion molecule (L1-CAM) promotes cell migration on fibronectin and laminin (Mechtersheimer et al., 2001).

Adhesion of migrating cells to their ECM substrate is also mediated by focal adhesions that connect the cellular actin cytoskeleton to their extracellular substrate *via* transmembrane integrins. These multiprotein structures depend on PM chol levels for their formation and dynamics. Chol depletion by mβCD has been shown to decrease α5β1 integrin-mediated cell adhesion to fibronectin, inducing changes in motility of lung adenocarcinoma L27 cells (Ramprasad et al., 2007). This could be explained by the role of membrane chol in the redistribution of GM3 ganglioside-associated α5β1 integrins in focal adhesion (Gopalakrishna et al., 2004). The loss of cell adherence induced by membrane chol depletion has been linked to the perturbation of focal adhesion dynamic and organization. In fact, Wang and coll. observed that lipid raft disruption inhibits focal adhesion disassembly due to excessive phosphorylation of paxillin and vinculin, two focal adhesion adaptor proteins (Wang et al., 2013). Moreover, mβCD inhibits phosphorylation of the focal adhesion kinase (FAK) in non-small cell lung cancer cell migration (Jeon et al., 2010). Mechanistically, LDL-chol are delivered to the PM together with activated FAK in close proximity of focal adhesions by the Rab8a-MyosinVb-actin pathway, favoring focal adhesions dynamics and cell migration (Kanerva et al., 2013; Takahashi et al., 2021). Altogether, those data point out the importance of membrane chol in the modulation of cell adhesions during cancer cell metastasis.

#### 4.2.4 Cholesterol-enriched domains and cancer cell invasion

Along with the adhesion to the surrounding substrate, cancer cells produce invadopodia to degrade the ECM, allowing them to progress in their environment. Invadopodia are actin-based membrane protrusions capable to enzymatically degrade the underlying ECM *via* recruitment and secretion of MMPs (Weaver 2006; Linder 2007; Gimona et al., 2008). Several studies have reported the importance of membrane chol for the formation and function of invadopodia (Figure 4B, left). In 2009, Caldieri and coll. showed in human melanoma cells that invadopodia display properties of chol-enriched PM domains and that the biogenesis and proteolytic activity of invadopodia depend on both appropriate PM chol levels and Cav-1, a regulator of chol transport to the PM (Caldieri et al., 2009). Those chol-enriched domains were further described as lipid rafts which are dynamically trafficked at invadopodia sites in breast cancer cells and are essential to establish those membrane protrusions (Yamaguchi et al., 2009; Nicolson 2015). Moreover, disruption of lipid rafts by mβCD-induced chol depletion suppresses breast cancer cell invasion by inhibiting invadopodia formation and expression of the membrane type proteolytic ECM-degrading enzyme MT1-MMP (Yang H. et al., 2016a). More recently, Maja et al. showed that malignant MCF10CAIa cells exhibit increased dorsal surface submicrometric chol-enriched domains which specifically contribute to cell invasion through Matrigel. Mechanistically, chol-enriched submicrometric domains can reach the ventral face where they control invadopodia maturation and ECM degradation (Maja et al., 2022).

### 4.3 Cell apoptosis

Another key hallmark of cancer cell is its resistance to cell death. Over the years, membrane chol has emerged as a key modulator of apoptotic signaling pathways, owing to its presence in lipid rafts. Besides survival proteins such as IGFR and EGFR, death receptors (i.e., tumor necrosis factor (TNF)-related apoptosis-inducing ligand receptor (TRAIL-R) and CD95/Fas) and downstream signaling molecules also aggregate and cluster in these specialized PM regions to induce apoptosis (Iessi et al., 2020; Mollinedo and Gajate 2020) (Figure 4A, right).

Upon binding of TRAIL, the cytoplasmic death domain of the transmembrane death receptors DR4 (TRAIL-R1) and DR5 (TRAIL-R2) recruits the adaptor molecule Fas-associated death domain (FADD) and the apoptosis initiating pro-caspases-8/10 to assemble the DISC (see Section 2.3). This complex allows auto-proteolytic activation of pro-caspases-8/10 that cleave and activate effector caspases-3/6/7 which cleave numerous intracellular targets activating the extrinsic apoptotic pathway, while caspase-8 stimulates the cleavage of BID activating the mitochondrial intrinsic apoptotic pathway (Johnstone et al., 2008; Ouyang et al., 2013). Aggregation of TRAIL death receptors within lipid rafts is mandatory for efficient cell death transmission (Marconi et al., 2013; Mollinedo and Gajate 2020). On the contrary, assembly of the TRAIL-DISC complex in non-rafts leads to caspase-8 cleavage inhibition and activation of ERK1/2, stimulating non-small cell lung carcinoma survival and proliferation (Song et al., 2007). Multiple studies have reported the correlation between DR5 function and its localization in chol-enriched domains (Merino et al., 2006; Gajate and Mollinedo 2007; Min et al., 2009). Moreover, Lewis and coll. showed that PM chol is necessary for DR5 dimerization and subsequent activity as chol depletion fails to induce caspase cleavage (Lewis et al., 2016). Resistance to TRAIL-induced cell death after membrane chol depletion by mβCD was also reported by several studies (Delmas et al., 2004; Marconi et al., 2013).

Binding of FasL/CD95L (Fas/CD95 ligand) to its receptor, the trimeric transmembrane death receptor Fas/CD95, stabilizes the latter and promotes oligomerization with adjacent Fas/CD95 trimers. The Fas-associated death domain (FADD) adaptor protein is then recruited near the receptors through homotypic interactions between their respective death domains, allowing pro-caspase-8 recruitment, formation of the DISC complex and execution of apoptotic signal transmission (Gajate and Mollinedo 2015; Levoin et al., 2020; Li et al., 2022; Mollinedo and Gajate, 2022). The antitumor ether lipid edelfosine has been found to induce apoptosis in leukemic cells through translocation of Fas/CD95, FADD and pro-caspase-8 into membrane rafts, demonstrating the involvement of lipid rafts in Fas/CD95-mediated apoptosis (Gajate and Mollinedo 2001; Mollinedo and Gajate 2006; Gajate and Mollinedo 2007; Gajate et al., 2009a). Interestingly, chol-targeting agents inhibit Fas/CD95 aggregation in rafts and apoptosis induced by edelfosine in multiple myeloma cells both *in vitro* and *in vivo* (Gajate and Mollinedo, 2001; Gajate and Mollinedo, 2007). This effect is reversible upon chol replenishment and can be mimicked upon ceramide addition which displaces chol from rafts (Mollinedo et al., 2010). For further details on edelfosine, see Section 5.4. Another study showed that chol depletion abolishes DISC formation and subsequent cell death of mouse thymocytes (Hueber et al., 2002). On the contrary, some studies showed that cellular chol depletion induces the spontaneous aggregation of CD95 in non-raft regions which promotes ligand-independent death signal transduction in various cell types (Gniadecki 2004; Bionda et al., 2008; Yi et al., 2009). Altogether, while lipid rafts are thought to act as scaffolds for the recruitment and activation of death receptor signaling pathway, thus constituting promising targets in cancer therapies, the specific mechanism remains to be clarified (Li et al., 2022; Mollinedo and Gajate, 2022).

### 4.4 Cell interaction with the microenvironment

Over the past few decades, it has become clear that interactions between cancer cells and their microenvironment are crucial for tumor progression and metastasis (Quail and Joyce 2013) and several studies reported the involvement of chol-enriched domains in the modulation of those interactions. This section aims to give an overview of current studies that have investigated the contribution of membrane chol in immune cell function and in mechanical responses of tumor cells towards acellular surrounding components.

#### 4.4.1 Immunity

Over the years, the complex interactions between the immune system and the tumor cells have been widely investigated. Within the tumor microenvironment, lipids play contradictory roles, which can support both anti-tumor immune response and pro-tumor immune response (Yu et al., 2021). Chol and its associated metabolites have been described to impact both innate and adaptive immunity (King et al., 2022). For examples, the oxysterol metabolite 27-hydroxycholesterol exerts pro-metastatic actions on breast cancer tumor by increasing the number of polymorphonuclear neutrophils and innate γδ T cells while decreasing cytotoxic CD8^+^ T cells (Baek et al., 2017). Upon membrane chol depletion in murine splenocytes, the activity of γδ T cells is impaired (Cheng et al., 2013). Interestingly, inhibition of chol esterification specifically increases chol content in the PM of CD8^+^ T cells, enhancing their function and proliferation (Yang et al., 2016c; Huang et al., 2020). Cancer cells-induced chol efflux from tumor-associated macrophages promote their reprogramming to an alternative immune-suppressive and pro-tumoral phenotype within the tumor microenvironment (Goossens et al., 2019; Sica et al., 2019).

Besides cellular chol and its metabolites, several studies have evidenced chol-enriched domains as modulators of both innate and adaptive immune responses (Katagiri et al., 2001; Varshney et al., 2016). In fact, lipid rafts serve as platforms for the recruitment and activation of immune receptors including single-pass membrane-spanning Toll-like receptors (TLRs) that act as primary sensors of pathogens (Kulkarni et al., 2021), FcεRI that mediates allergic response in mast cells (Sheets et al., 1999; Varshney et al., 2016) and B- and T-cell immune receptors involved in adaptive immune response. These latter were indeed shown to translocate to lipid rafts upon antigen binding where they are respectively phosphorylated by the Src-like tyrosine kinase protein Lyn and by the lymphocyte-specific protein tyrosine kinase Lck, leading to B and T cell activation and subsequent activation of the signaling cascade (Xavier et al., 1998; Cheng et al., 1999; Gupta and DeFranco 2003; Beck-Garcia et al., 2015; Dinic et al., 2015) (Figure 4C). However, while many investigations revealed a role of chol-enriched domains as modulators of immune responses, literature fails to report if and how chol dysregulation in cancer alters the signaling of immune cells.

#### 4.4.2 Mechanical responses

It is known that the rates and routes of metastatic dissemination strongly depend on the biochemical composition and biophysical characteristics of the surrounding ECM which is remodeled from normal to tumor tissues (Yuzhalin et al., 2018; Winkler et al., 2020). The specific protein composition of the ECM provides an anchorage for adjacent cells and modulates signaling transmission by interacting with specific cell surface receptors (Theocharis et al., 2016; Nazemi and Rainero 2020; Popova and Jucker 2022). We already described the role of several adhesion molecules in cell migration, invasion and adhesion **(**see Section 4.2) as well as their dependence to chol-enriched domains. More information on how these molecules interact with the ECM to regulate intracellular signaling networks can be found in literature reviews (Kim et al., 2011; Gasparski and Beningo 2015; Hastings et al., 2019; Sainio and Jarvelainen 2020).

Besides its biochemical composition, the ECM biomechanical properties are remodeled under the influence of surrounding tumor stromal and cancer cells. These ECM biophysical properties include stiffness, viscosity, density, topography and porosity and directly force cancer cells to respond to those sensed ECM alterations by transducing adaptation signaling leading to enhanced tumor progression. For example, cancer cells adapt to increased ECM rigidity stimulus by producing mature and proteolytically active invadopodia (Gasparski et al., 2017). In fact, cancerized ECM is stiffer than normal ECM and several studies reported that invadopodia formation, maturation and activity are enhanced on stiffer ECM compared to softer substrate (Alexander et al., 2008; Parekh et al., 2011; Aung et al., 2014; Najafi et al., 2019; Berger et al., 2020). Moreover, tumor tissues are characterized by elevated viscosity through which cancer cells migrate in a protease-independent manner, by widening and lengthening invadopodia protrusions to physically open up a channel in the plastic matrix (Wisdom et al., 2018). This not only suggests that invadopodia adapt to the biomechanical cues from the microenvironment but also that they act as mechanosensors to facilitate cancer cell progression (Masi et al., 2020). Invadopodia depend on membrane chol-enriched domains for their formation as well as MMP-mediated proteolytic activity (Caldieri et al., 2009; Yamaguchi et al., 2009; Nicolson 2015; Maja et al., 2022) as discussed in Section 4.2.

### 4.5 Extracellular vesicle release

EVs are particles released by cells in the extracellular medium. Those are generally classified into three groups, exosomes, microvesicles (MVs) and apoptotic bodies (Gyorgy et al., 2011; Turturici et al., 2014; Raposo and Stoorvogel 2013; Sedgwick and D'Souza-Schorey 2018).

In recent years, research on exosomes has increased, especially for their role in cancer progression and metastasis as well as their potential use in cancer therapy (Tai et al., 2018; Osaki and Okada 2019; Dai et al., 2020; Liu et al., 2021). The role of membrane lipids in exosome biogenesis goes beyond the scope of this manuscript as exosomes do not originate directly from the PM. Readers can refer to (Pfrieger and Vitale 2018; Verderio, Gabrielli, and Giussani 2018) for more information on how chol and sphingolipids contribute to exosome formation and function.

We will here focus on MVs and on the role of chol-enriched domains in their production and regulation. Ranging from 100 nm to 1 μm in diameter, MVs are small lipid bilayer-bound EVs generated by local deformation and budding of the PM. Several evidences support a role of PM chol or chol-enriched domains in MV biogenesis and shedding (Figure 4B, right). For instance, MVs are enriched in the lipid-raft associated tissue factor (TF) and in the chol-binding protein stomatin (Salzer et al., 2002; Del Conde et al., 2005; Salzer et al., 2008). As another example related to cancer, MVs released from murine leukemia cells are enriched in chol and SM (van Blitterswijk et al., 1979). Moreover, PM chol depletion by mβCD of human THP-1 monocytes impairs membrane shedding and leads to reduced MV abundance, while chol enrichment of the same cell line stimulates MV release (Del Conde et al., 2005; Liu et al., 2007). Mechanistically, based on the vesiculation of chol-enriched domains upon RBC storage at 4 °C, theoretical work and biophysical experiments on model membranes, the line tension at domain boundary has been proposed as driving force for lipid domain vesiculation (Lipowsky 1992; Baumgart et al., 2003; Yang et al, 2016b; Leonard et al., 2017b).

## 5 Targeting membrane cholesterol and cholesterol-enriched domains as a promising strategy in cancer

**FIGURE 5:**
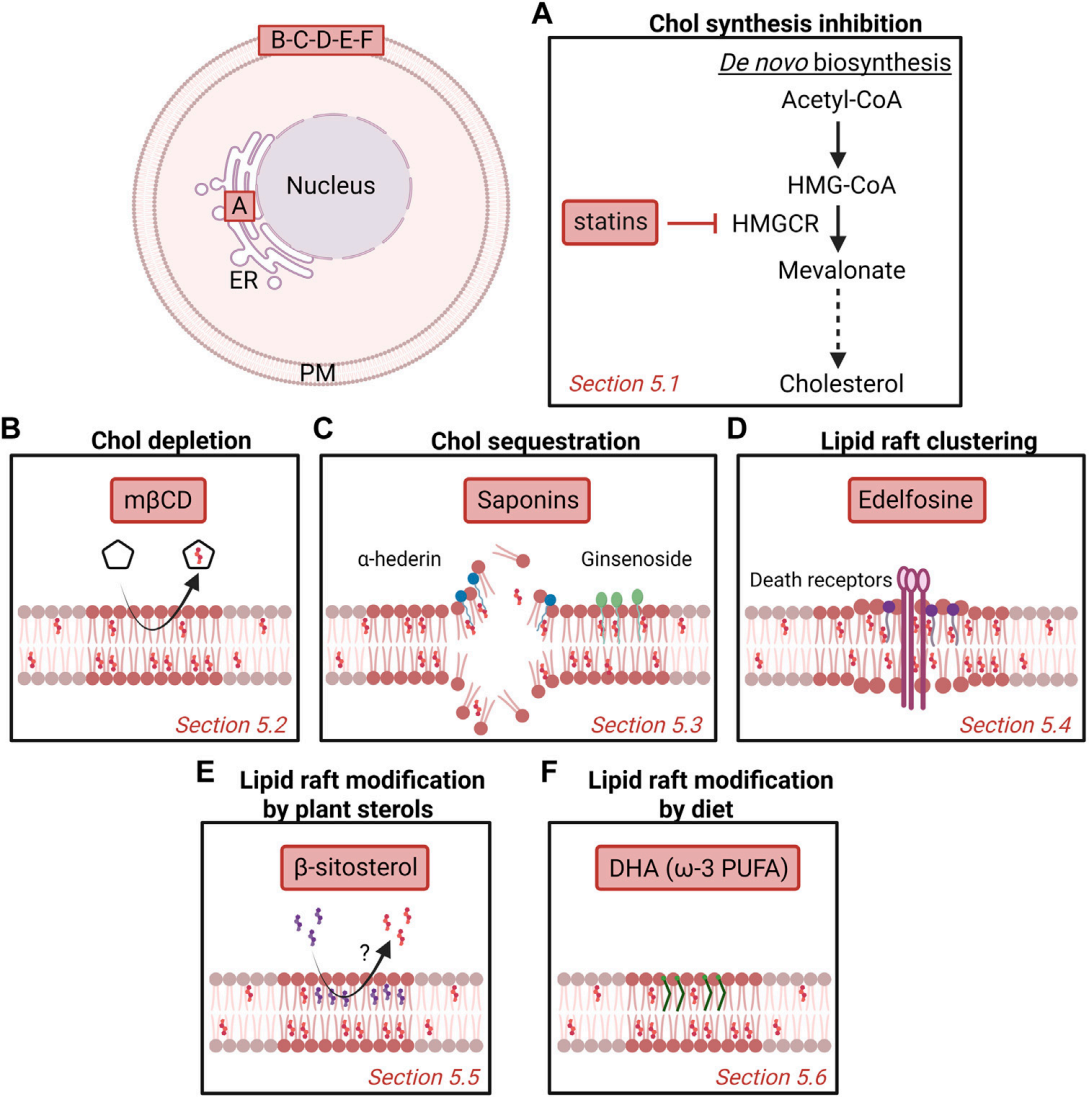
Potential strategies to target cholesterol content and membrane domains enriched in cholesterol as anti-cancer therapy. Several strategies have been proposed over the years, including inhibition of chol synthesis in the endoplasmic reticulum (ER; A) or targeting plasma membrane (PM) chol (B-F). Nevertheless, more investigation is needed to understand the mechanism behind as well as the potential benefit as anticancer drugs for patients. **(A)** Statins inhibit the 3-hydroxy-3-methylglutaryl-coenzyme A reductase (HMGCR) that catalyzes the conversion of HMG-CoA to mevalonate. **(B)** Methyl-β-cyclodextrin (mβCD) extracts membrane chol and thereby impairs chol-enriched domain organization and integrity. **(C)** Saponins can disrupt chol-enriched domains and induce membrane pore formation. **(D)** Edelfosine interacts with lipid rafts and induces the recruitment and clustering of Fas/CD95 death receptors. **(E)** Plant β-sitosterol has been proposed to modify the composition and stability of lipid rafts. **(F)** The fish oil-derived polyunsaturated fatty acid (PUFA) docosahexaenoic acid (DHA) modifies lipid raft composition, size and clustering capacities. See the Section 5 of the text for further details.

As chol-enriched domains seem to actively participate to the phenotype of malignant cells, they could represent promising targets for anticancer treatment. From directly targeting chol metabolism or its membrane content/distribution to adjusting sterol intake, diverse strategies have been developed over the years. In this section, we summarize the key recent findings on the potential anticancer benefit of targeting chol content (Sections 5.1-5.3) and its clustering into rafts (Sections 5.4, 5.5). Finally, we propose additional strategies such as PUFA and ceramide to target other lipids than chol (Section 5.6) (Figure 5).

### 5.1 Cholesterol synthesis inhibition

Statins are competitive inhibitors of HMGCR, the enzyme that catalyzes the conversion of HMG-CoA to mevalonate (Figure 5A). By inhibiting the mevalonate pathway, statins reduce the amounts of end-products such as chol, explaining why they were first used in the treatment of dislipidemic disorders (Endo 2010).

Over the years, many compounds have been developed, including mevastatin, lovastatin, simvastatin and pravastatin, and the extension of their use in anticancer therapy has been proposed (Farwell et al., 2008). In fact, many clinical studies reported the statin anti-tumor effects and showed that, whether used in monotherapy or in combination with chemotherapeutic drugs, statins efficiently reduce the occurrence of many types of cancers and cancer mortality ratios (Gbelcova et al., 2008; Sassano and Platanias 2008; Nielsen et al., 2012; Fatehi Hassanabad 2019). Statins were indeed shown to impact cellular processes such as proliferation, apoptosis, angiogenesis and metastasis, both *in vitro* and *in vivo* (reviewed in (Sopkova et al., 2017)).

However, while statins are perceived as new ally in the quest towards a cancer-free world, it is important to note that (i) those drugs induce moderate-to-severe side effects (Thompson et al., 2016), (ii) a significant proportion of patients show statin intolerance (Alonso et al., 2019), and (iii) statin antitumor effects are highly dependent on the type of cancer (Sopkova et al., 2017). This emphasizes the need to optimize current statin-based therapy protocols and to develop new specific chol-targeting anticancer drugs (Gu et al., 2019).

### 5.2 Cholesterol depletion

The importance of PM chol content for cancerous processes makes it an interesting target of compounds able to extract chol from cell membranes such as cyclodextrins. Among those, mβCD has been proposed to present a anticancer activity in a number of cancers. In fact, at the optimal concentration, mβCD preferentially extracts membrane chol and impairs chol-enriched domain organization and integrity, leading to perturbed signaling in cancer cells (Mollinedo and Gajate 2020; Vona et al., 2021) (Figure 5B).

However, care must be taken because of mβCD drawbacks. First, mβCD is not specific to chol as high mβCD concentrations were shown to extract other hydrophobic molecules from the PM such as fatty acids (Brunaldi et al., 2010). Second, mβCD treatment would require cancer cells to exhibit higher surface chol content than their healthy counterparts. We showed that it is indeed the case in two invasive breast malignant cell lines, the MCF10CAIa and the MDA-MB-231 (Maja et al., 2022). Nevertheless, it remains to evaluate whether this observation could be generalized to other breast cancer cells lines and in other types of cancer. Third, the cytotoxic effect of mβCD depends on its concentration and on the cancer type studied (Li et al., 2006).

Nonetheless, the use of mβCD in combination with some anticancer drugs has been shown to increase their efficacy in various cancers, either by perturbing PM permeability which sensitize the cells to the chemotherapeutic drug (Upadhyay et al., 2006; Mohammad et al., 2014), or by using the cyclodextrin as anticancer drug delivery system (Tian et al., 2020; Bai et al., 2021).

### 5.3 Cholesterol sequestration

Besides chol depletion, chol sequestration represents another potential chol-based anticancer strategy. Among potential drugs, one can cite the saponins, a heterogeneous group of sterol and triterpene glycosides naturally found in plants and widely used in medicine for their various pharmacological properties including anticancer activities (Lorent et al., 2014; Xu et al., 2016; Elekofehinti et al., 2021). Besides digitonin and α-hederin, one can cite the Ginseng saponins, also called ginsenosides (Figure 5C). Total ginsenosides of Chinese ginseng are able to induce cell cycle arrest and apoptosis on colorectal carcinoma HT-29 cells (Li et al., 2018). Studies on individual ginsenosides have revealed that the anticarcinogenic effects result from different mechanisms involving the alteration of lipid rafts, pore formation and modulation of reactive oxygen species production. In addition, some ginsenosides inhibit drug efflux pumps, which can enhance the activity of conventional chemotherapeutic agents. For a review on this topic, please refer to (Verstraeten et al., 2020).

Despite the increasing amount of research on saponin characterization for their anticancer properties, low bioavailability and toxicity remain challenging obstacles that would have to be overcome in order to optimize the drug for clinical trial (Elekofehinti et al., 2021).

### 5.4 Lipid raft clustering

The co-clustering of lipid rafts and Fas/CD95 death receptor can be induced by a number of molecules, promoting thereby apoptosis (reviewed by (Mollinedo and Gajate 2020)). Among those, the antitumor alkyllysophospholipid edelfosine can be considered as the lead compound. Edelfosine interacts with high affinity with PM chol and is able to destabilize synthetic membranes (Ausili et al., 2018). In cells, it is able to accumulate in and disorganize rafts by increasing PM thickness and fluidity, subsequently inhibiting the PI3K/Akt proliferation signaling pathway while promoting Fas death receptor recruitment and apoptosis of cancer cells (Ausili et al., 2008; Hac-Wydro et al., 2011; Castro et al., 2013; Mollinedo and Gajate 2015; Ausili et al., 2018) (Figure 5D). For instance, treatment of human acute T-cell leukemia Jurkat and acute myeloid leukemia HL-60 cells with edelfosine induces apoptosis through the recruitment and clustering of Fas/CD95 death receptor in lipid rafts (Gajate and Mollinedo 2001). Subsequent studies demonstrated that edelfosine induces the co-clustering of Fas/CD95 and rafts in different types of cancer cells (Gajate et al., 2004; Gajate and Mollinedo 2007; Gajate et al., 2009b). In support of the role of rafts in the edelfosine-induced apoptosis, raft disruption by chol depletion with mβCD and chol sequestration with filipin block edelfosine-induced Fas/CD95 aggregation and apoptosis. Moreover, ceramide addition in multiple myeloma cells displaces chol from rafts and inhibits the apoptotic response induced by the antitumor ether lipid (reviewed in (Mollinedo and Gajate, 2022)).

Altogether, those data indicate that edelfosine represents an interesting therapeutic approach. However, its toxicity and modest efficacy makes it unlikely to be used in patients, contrarily to its analog perifosine (Alves et al., 2016). The 10-(Octyloxy) decyl-2-(trimethylammonium) ethyl phosphate (ODPC) is another alkyllysophospholipid that has been shown to target chol-enriched domains in model membranes and induce apoptosis of leukemia cells (Thome et al., 2012; Alves et al., 2016).

### 5.5 Lipid raft modification by plant sterols

β-sitosterol is a bioactive phytosterol naturally found in plant cell membranes, with a chemical structure similar to that of chol except for an additional ethyl group at C-24. β-sitosterol possesses various biological actions including antidiabetic, antihypercholesterolemic, antimicrobial, antioxidant, immunomodulatory and anticancer (Ling and Jones 1995; Babu and Jayaraman 2020; Bin Sayeed and Ameen 2015). For instance, it inhibits breast cancer cell invasion and adhesion (Awad et al., 2001), is associated with human prostate cancer cell cycle arrest at the G2/M phase (Awad et al., 2005), specifically inhibits the proliferation of colon cancer cells but not normal cells (Jayaprakasha et al., 2007), and reduces SM membrane content to the benefit of ceramides (Awad et al., 1998; Codini et al., 2021). Interestingly, β-sitosterol, was shown to incorporate in breast cancer cell membrane where it promotes apoptosis by activating Fas signaling, a lipid raft-dependent apoptotic pathway (Awad et al., 2007).

This suggests that β-sitosterol could counteract the pro-tumorigenic activity of chol by modulating PM composition and/or biophysical properties (Figure 5E). This hypothesis is supported by the observation that β-sitosterol weakens the interactions between molecules on chol/SM/GM3 mixtures and decreases film stability and condensation (Hac-Wydro 2013). Nevertheless, more investigation is necessary to evaluate the potential benefits of β-sitosterol as an anticancer drug for patients (Novotny et al., 2017).

### 5.6 Additional lipid-based strategies

Diet intervention appears as an interesting indirect strategy to target membrane chol in cancer. Dietary fatty acids have been shown to modulate membrane lipid composition and function and their role has been suggested in cancer prevention (Berquin et al., 2008; Preta 2020). Specifically, the docosahexaenoic acid (DHA), a fish oil-derived PUFA, has been described to alter both PM lipid composition and properties by targeting two essential lipid raft components, chol and SM (Fan et al., 2003; Fan et al., 2004) (Figure 5F). In consequence, the signaling network that relies on lipid raft integrity is impacted due to modification of the raft composition, size and clustering capacities as well as alteration of membrane fluidity, ordering and permeability (Turk and Chapkin 2013; Levental et al., 2016; Wassall et al., 2018; Bie et al., 2020). Studies demonstrated on a wide number of cancer cell lines and preclinical models that DHA is able to inhibit cell proliferation and growth (D'Eliseo and Velotti 2016; Newell et al., 2017). However, the mechanisms by which DHA acts on cancer cell cycle regulation is not yet fully understood. Ultimately, elucidating the real benefits of dietary PUFA in cancer is a prerequisite step to determine if PUFA could be used in the future in combination with current chemotherapy.

Targeting the ceramide may be an alternative/complementary strategy, especially for radiation-resistant cancer cells. Indeed, ceramides or synthetic metabolically stabilized analogs with pro-apoptotic properties (like C16-ceramides) have been proposed as useful anticancer agents (Blaess et al., 2015). Studies have demonstrated that increases in cellular ceramide result in cell death induction and radiosensitization of prostate cancer tumors and that exogenous C6- and C16-ceramide treatment delays the growth of prostate cancer spheroids and induces cell apoptosis (Ketteler et al., 2020; Samsel et al., 2004).

## 6 Conclusion and future challenges

In this review, we have highlighted that chol levels and chol-enriched domains could represent a promising target in cancer therapy based on their implication in cell survival/proliferation, migration/invasion and apoptotic signaling *a. o.* (Section 4). However, although diverse strategies have been developed over the years, all present more or less substantial limitations related to modest efficacy, moderate-to-severe side effects, intolerance and cancer type-dependent antitumor effects (Section 5).

Those data emphasize the need to optimize current chol targeting strategies, to develop new specific chol-targeting anticancer drugs and/or to combine them with additional strategies targeting other lipids than chol, as discussed in Section 5.

Those objectives can only be achieved if we first understand the mechanisms that govern the formation and deformation of the different types of lipid domains and their interplay in healthy cells (Section 2.3). Indeed, despite enormous progress during recent decades, various crucial questions remain to be addressed, including: (i) what is the exact size, diversity and interplay between different types of domains; (ii) to what extent do nanometric rafts coalesce into submicrometric domains under appropriate conditions; (iii) what is the lipid and protein composition of lipid domains; (iv) is there a correspondence between lipid domains at outer and inner PM leaflets; (v) how can energetic considerations, intrinsic membrane factors and extrinsic factors be integrated to regulate domains; and (vi) what are the physiological roles of lipid domains.

From there, the next step will be to continue to evaluate whether and how some of these mechanisms are deregulated in cancer, whether observations can be generalized in different types of cancer and whether some specific features can be highlighted in cancer vs*.* non-cancer cells as promising strategy for anticancer therapy. As highlighted in Section 3, data on (i) chol composition and membrane biophysical properties, (ii) chol-enriched domain abundance and properties, and (iii) chol transversal asymmetry in cancer cells are provided in the literature but they are sparse, usually based on indirect evidences and sometimes contradictory. As much as this research was unthinkable a few decades ago, the recent development of new lipid probes and innovative imaging techniques should make it possible to reach these objectives.

## Abbreviations

ABC, ATP-binding cassette; ACAT, acyl-Coenzyme A:cholesterol acyltransferase; Cav-1, caveolin-1; CE, cholesteryl ester; Chol, cholesterol; DISC, death-inducing signaling complex; ECM, extracellular matrix; EGF, epidermal growth factor; ER, endoplasmic reticulum; EV, extracellular vesicle; FcεRI, high affinity immunoglobulin E receptor; GPI, glycosylphosphatidylinositol; HMGCR, HMG-CoA reductase; IGF, insulin-like growth factor; LDL, low-density lipoprotein; LXR, liver X receptor; mβCD, methyl-β-cyclodextrin; MMP, matrix metalloproteinase; MV, microvesicle; PC, phosphatidylcholine; PE, phosphatidylethanolamine; PI3K, phosphatidylinositol 3-kinase; PIP2, phosphatidylinositol-4,5-bisphosphate; PIP3, phosphatidylinositol-3,4,5-trisphosphate; PM, plasma membrane; PS, phosphatidylserine; PUFA, polyunsaturated fatty acid; RBC, red blood cell; SM, sphingomyelin; TRAIL, tumor necrosis factor-related apoptosis-inducing ligand.
